# Book Reviews

**Published:** 1819-11

**Authors:** 


### CRITICAL ANALYSIS

OF

### RECENT PUBLICATIONS, IN THE DIFFERENT BRANCHES OF MEDICINE AND SURGERY.

"I would have men know, that, though I reprehend the easie passing over of the causes of things, "by ascribing them to secret and hidden vertues and properties; (for this hath arrested and laid "asleepe all true enquiry and indications;) yet I doe not understand but that, in the practical "part of knowledge, much will be left to experience and probation, whereunto indication cannot "so fully reach: and this not only in *specie*, but in *individuo*. Yet it was well said, *Vere scire* "*esse per causas scire*."—BACON.

***Elements of Medical Logick, illustrated by practical Proofs and Examples; including a Statement of the Evidence respecting the Contagious Nature of the Yellow-Fever.*** By SIR GILBERT BLANE, Bart. Fellow of the Royal Societies of London, Edinburgh, and Gottingen; Member of the Imperial Academy of Sciences of St. Petersburgh; and Physician to the Prince Regent. 8vo. pp. 219. London: T. and G. Underwood. 1819.

[*Concluded from p.* 314.]

THERE can be no question more interesting to the young physician of liberal acquirements and a reflective disposition of mind, than that relating to the importance of physiological and other scientific knowledge, in its application to medicine as an art. The history of the revolutions of medical opinions in different ages, and of their diversity at the present period, will rather add to his embarrassment than lead him to any determinate mode of conduct.

The method of HIPPOCRATES, so generally approved by experienced physicians, he will find to have been strictly enlightened empiricism. Many things, however, tend to make him not regard this in a favourable manner: he is unwilling to believe that the greater part of his laborious scholastic studies can be of so little utility to him as that method would inculcate; and he turns with eagerness to the opinions of the dogmatists, so flattering to his own inclinations. But let him pass in review the most brilliant systems of the methodic sect, whose precepts they have followed, and contemplate the results of the application of those of ERASISTRATUS, GALEN, PARACELSUS, BOERHAAVE, HOFFMANN, CULLEN, and BROWN; and he will be in doubt whether the benefits some of those have conferred on human nature, have not been counterbalanced by the ills that have emanated from others. The doctrines of another sect, especially if he be disposed to metaphysical reflection, will then appear in the most glowing colours. Its votaries will tell him, that the human body, which possesses the power of its own preservation;—of appropriating to its use aliment and air;—of resisting the destructive influence of various external agents, and of re-producing parts that have been destroyed by others;—may be well supposed to be able, in most instances, to restore, without the aid of art, the harmony of its actions, when accidental circumstances have thrown them into disorder. To observe the progress of the actions necessary to effect this through their different revolutions, without attempting to interfere with them when they proceed in a proper manner; and to regulate them, if they casually deviate from it, by such means as experience may have shown to be successful in similar cases, is, then, considered to be the only proper conduct of the true physician. But, there is only one step from this to absolute Pyrrhonism; the gloomy and repulsive regards of which will probably drive him from the paths of the *Naturists,* although they have been adorned by almost the finest genius that ever walked in the regions of medical science. The *Eclectics* will point out the course of SYDENHAM, BARBEIRAC, and BAGLIVI, to his view; but he will sometimes be obliged to turn aside from them, and will then, notwithstanding the assurances of his advisers, often find himself in the midst of a mere chaos, constituted of the dispersed ruins of other systems.

Has not medical knowledge, then, attained such a state as to admit of some conclusions on this subject, that may serve as general principles for the conduct of the young physician? This interesting question is discussed in the second section of the work before us.

In order to preserve a connected view of the subjects of this treatise, we should advert to the physiological dissertations that have already engaged our attention; or, from the want of any application of them, it might appear that our analysis had not preserved an unbroken train. The following paragraph will exculpate us in this respect, though it will probably lead the reader to criminate us in others; since we have not been able to discern the peculiar view to which the author therein alludes:

"The author has thus endeavoured to enumerate and define the primary elements, or ultimate facts, belonging exclusively to animated nature. In an attempt which is new,—in a subject, of which he has taken a view peculiar to himself,—he does not dare to think that he has attained any thing like perfection. It is evident, however, that it is only by following out an analytical scheme of this kind, that a foundation can be laid for the genuine principles of theoretical medicine; for the elements of disease can only be expounded by a thorough knowledge of the elements of life and health. But it is not his intention now to apply it synthetically, by building upon it any system of nosology, physiology, or pathology; far less to bewilder himself or his reader, by agitating the question, whether life consists in the play of those principles, or if it is something superinduced on them. His sole object, in this analysis, is to convey an adequate conception of the great difficulties which those have to encounter, who would found practical medicine on a knowledge of the animal economy; and to bespeak a liberal indulgence for the errors of those, who, in attempting this, have had to grope and wander in more dark and intricate mazes than what has fallen to the lot of any [other] class of enquirers into the various departments of nature."

We repeat, we have not been able to discern the author's peculiar view in the preceding dissertation, either as relates to physiological principles or the pathological application of them; except the following remark, relating to the *restorative energy*, may be considered to be a glimpse of it. We retrace our steps to adduce it.

"If one of the morbid poisons exciting fever, should assail life by attacking one of its fundamental principles, the generating power of heat, this principle may re-act with such violence, as to make it one of the main objects to repress it, either by internal remedies, or by the external application of cold; and the converse of this will happen, should the re-action be too feeble. This is not to be considered as an adopted theory, but merely a matter of hypothetical illustration."

The reader will, we hope, excuse our making any remarks on this notion, and not consider that, in avoiding it, we shrink from the performance of our duty. The author promises to advert to this subject under another head; but we have not been able to discover where he has done this, unless the query, whether the *determination* to particular organs may not be considered "as a function by itself," dependant on the motive energy, may be thus regarded.

We now return to the discussion on the utility of anatomical and physiological knowledge in the practice of medicine. The author first adverts to the errors that have arisen from the attempts made to explain the functions of animal bodies by the application of chemical and mechanical laws; and then takes a partial view of the circumstances that in different ages have either favoured, or retarded, the progress of practical medicine. In the rude state of society, he remarks, this seems to have been more indebted to those who discovered active and useful medicines, than to the labours of the learned. He passes a tribute of praise to Democritus and Hippocrates; and to Aristotle, as a "most diligent observer of nature and collector of facts;" but, he continues, the syllogistic logic of this philosopher soon usurped the place of all literary and scientific pursuits. Yet Sir Gilbert Blane acknowledges, that, "in those dark times, [the middle ages,] it was better than no knowledge at all; and I am one of those who are of opinion, that this logick, though it affords little or no assistance in the discovery of practical truths, and the interpretation of nature, *is yet an excellent discipline of the mind, tending to give precision to language and thought;* more, indeed, in moral science and literature, than in the physical and exact sciences; *and that some knowledge of it can hardly be dispensed with in a liberal education.* I am thankful that it made part of the academical education which I received."

We have been particularly pleased with the sentiments in the above paragraph which we have taken the liberty to transcribe in italic characters, and wish Sir Gilbert Blane had expressed his opinion respecting the mathematics in a similar point of view: we think he must equally approve of the study of them; and they are now strangely neglected by physicians in general. MEAD said, he hoped to find this species of knowledge become thought as necessary to a physician as the acquisition of the Greek and Latin languages; but, when the student reads his treatise on the Bite of the Tarantula Spider, and perceives the use that physician made of it, he must be disposed to treat his opinion on this subject with contempt: and the authority of Hippocrates and Galen will probably not be regarded with much more respect.

The merits of BACON are next alluded to, and appreciated: Sir Gilbert Blane, however, objects to his opinions respecting the utility of searching after first causes; but we never before saw them regarded in the manner in which he seems to view them.

Medicine, however, he continues to observe, did not avail itself of the light which broke upon science in general in the seventeenth century; but rather, from the wrong application of the physical sciences, became turned from its course, and oppressed with new errors. Even the knowledge of the circulation of the blood, "by its perverted application, tended to corrupt and mislead, by a loose adoption of the principles of mechanical philosophy, so well laid down in that age by Galileo and others."

We cannot have a more forcible illustration of this, than the manner in which the determination of the fluids to particular parts was for a long period regarded, and is indeed at the present time, by many physicians. The powerful influence of the nervous system in regulating the circulation, the peculiar action of the capillary arteries, and the distinct circles, as it were, formed by particular organs, have by them been lost sight of; and, consequently, with this, the true knowledge of the real nature and origin of numerous diseases.

The well-known errors of the mechanists are then brought into view, especially those of BOERHAAVE; though the author does not neglect to pay a just tribute to the talents and acquirements of that celebrated medical reformer. But here Sir Gilbert Blane proceeds in too rapid a manner; since he passes over unnoticed the true founder of the present system of physiology to arrive at CULLEN, who, he says, "first clearly marked, and defined, the principles of life, as distinguished from those of dead matter."

Let us indulge in some remarks on this subject. We shall apologize for these, and some other circumstances respecting our conduct in the review of this work, before we conclude.

GLISSON may be considered as the legitimate founder of the present doctrine of vitality. He announced that matter is not wholly *inert*, as almost all the moderns who followed the doctrines of PLATO had believed. He supposed it to be essentially active; and that the properties thus given to it by the Author of nature, were the cause of the phenomena we observe, not only in the great bodies of the universe, but also in chemical changes, and in the re-actions of organized bodies on external agents. This doctrine he applied in a particular manner to the functions of the animal economy; and gave the term *irritability* to the power which organs derive from the active properties of the matter of which they are composed.* LEIBNITZ and HOBBES supported these opinions; and GORTER afterwards extended the application of them to plants.†

It remained for HALLER fully to develope and illustrate these principles, in such a manner as to leave no doubt respecting their truth in the mind of any man who has well examined them, and who will "on reason build resolve." Nothing remained to be effected but the discovery of some of the more minute relations of the phenomena to which they give rise; and, since his labours, they have formed the basis of physiology. The theory thence formed was applied to pathology with the utmost precision by BORDEU, who flourished before CULLEN; though the latter, from his situation as a professor in one of the most celebrated schools of Europe at that period, gave it the *eclat* necessary to lead to its general adoption.

Many physicians have expressed doubts of the utility of this sort of knowledge to the art of medicine; and Sir Gilbert Blane thinks them worthy of so much attention, as to be made the subject of a formal examination. After some general reflections on this subject, he proposes the following query:

"Are we, then, to admit, that the greater part of what we have been taught at the schools of physic, and of what we have read, or may read, in books, is in no wise conducive to our practical improvement?"

Far from it, he replies,—for

"Though anatomy, physiology, and pathology, should be proved

* See his Treatise *De Ventriculo et Intestinis*; 12mo. 1677.

† *Excercitationes Medicæ Quatuor*; Amstelodamiæ, 1737.

to be of little or no avail,—nay, of pernicious tendency, in the practice of physic, the acquisition of these branches of education is nevertheless indispensable, in order to be armed with antidotes against the influence of fallacious theories, and to obtain the guidance of true beacons, instead of false lights. There is nothing better known to those who are conversant in medical practice, than that the most ignorant and shallow, those of the least learning,—nay, those of no learning at all,—are the most addicted to hypothetical reasoning, the most infected with presumption and conceit.

"The knowledge of nature in all its branches," he also observes, "is an indispensable requisite in the cultivation of the mind. It is highly useful, were it only as a gymnastic exercise of the understanding, by that salutary discipline of the mental faculties, implied in the acquisition of habits of attention, and the practice of the reasoning powers. Besides, all arts and sciences have a bearing on each other; and the history and philosophy of animal life, is surely as necessary an accomplishment to a physician as any other branch of science or literature."

It also, Sir Gilbert Blane says, tends to free the mind from superstition, which has exerted so much deleterious influence in the practice of medicine.

The concluding reply is somewhat more favourable to the cause of physiological knowledge, though much of its force must be averted by the reflections contained in those which have preceded it.

"It must be obvious to every reflecting mind, that those who have made themselves acquainted with the various organs and functions engaged in the animal economy, must have a great advantage in practice over the unlearned empiric, in discriminating the morbid affections from each other, and in varying accordingly the respective means of relief. For instance, a physiologist and anatomist, from his knowledge of the intimate nature of morbid affections, the difference of their seat, and other circumstances, is able to distinguish spasmodic from inflammatory pains; a distinction which would not readily occur to an uncultivated observer, but of the most vital importance in practice; for the remedies required for the relief and cure of a spasmodic pain in the stomach and bowels, demands a treatment not only different, but opposite, to that which proceeds from inflammation. It is only anatomical and physiological science that can give a practitioner a clear and vivid conception of these and other distinctions essential to the safe and efficient treatment of diseases.

"Whatever doubts there may be with regard to the degree in which anatomy is useful in physic, there can be no doubt of it with regard to surgery, in which an accurate knowledge of the relative position and structure of organs is indispensable."

"But," the author then observes, "if the benefits derivable to medicine from physiological science are so limited, from what other and better source is improvement to arise? The answer is, from accurate observation: in other words, from enlightened empiricism." Of this Sir Gilbert Blane gives a well-drawn and impressive character, though it is rendered a little abstruse by his peculiar opinions respecting the responsiveness of our mental faculties to the constitution of external nature. We transcribe from it the following passage, which contains some notions, that, to say the least of them, are ingenious, and certainly merit the attention of those who are disposed to philosophical reflection.

"The avoiding of fire, of precipices, the collision of hard and pointed bodies, may be quoted as examples of this [the responsiveness of our mental faculties to the constitution of external nature]. And what is called sagacity, in the adult ages of life, is a sort of approach to, or imitation of, this intuitive faculty; but, instead of being the immediate suggestion of nature, is founded on cultivation, which, by practice, learns to connect cause and effect, means and ends; an operation which, in well-turned minds, is performed with promptitude and precision, by interpreting fairly the appearances of nature, and stripping them of those adventitious fallacies which mislead ordinary minds. In order to attain this, there are required an appropriate natural capacity, the good fortune of not being beset with prejudices in early life, an habitual exercise in the observance of nature, a candid and ingenuous disposition, an ardent love of truth, an exalted sense of duty, a large store of facts in a correct and tenacious memory, the power of combining, comparing, and discriminating, these, by an intuitive glance, in the moment of applying them to the practical end in view. This is what is understood by the term *tact* in English and French, and εὐστοχία in Greek; being the faculty by which practical facts are decided on, and is performed by an instantaneous, silent, and almost unconscious, calculation and induction, to be met with only in minds at once happily constituted and highly cultivated."

We have now only to adduce the author's general conclusion, in order to complete our analysis of this part of the work. We shall give this in his own words.

"From all that has been said, we ought to be in some measure qualified to come to a decision on the celebrated question of the comparative merits of the empirical and dogmatical methods of cultivating physic. It seems pretty evident, that, if either method were employed exclusively, or carried to an extreme, the art of physic would suffer, both in its efficiency and its prospects of future improvement. It has clearly appeared, that, under such a complication of causes, influencing the operations of life, it would be utterly hopeless to decide any point purely and strictly *à priori;* and that it is absolutely necessary that experience be called in as an aid and a test to the inferences of theory. On the other hand, a blind empiricism would be found deficient, without that discriminative judgment, founded on an acquaintance with the laws of life, and without those enlarged and correct views of general nature, by which the excess of credulity and scepticism is equally repressed."

Having thus considered the chief of the obstacles to the progress and improvement of medicine, and the errors and abuses of false or misapplied theory, the author proceeds to that of the rest: these he supposes to be, *the great diversity observable in the constitution of individuals; the difficulty of appreciating the efforts of nature, and of discriminating them from those of art; superstition; the ambiguity of language; and the fallacy of testimony.*

The varieties in the character of disease arising from an identical cause, and the diversity in the operation of certain remedies, exemplified particularly in that of purgatives, form the subjects for illustration of the first of the above series; and this section concludes with questioning whether it would be too much to affirm, that all the practical works in existence ought to be re-composed, in order to insert in them, for the benefit of mankind, and the credit of the profession of physic, the following qualifying words: "The practice recommended will be found to answer in a great majority of cases; but there are numerous exceptions to it, which it behoves every judicious and conscientious practitioner to bear in mind."

On the next subject the author makes some general remarks only; but they bear the character of the rest of the moral reflections comprised in this work, of which we shall hereafter speak. Of the influence of superstition, he alludes to several remarkable instances; as, the unresisting submission to the progress of the plague of the Mahomedans; the opposition to inoculation of the small-pox from religious prejudice; the belief in the powers of magic, amulets, &c. in the cure of diseases, many curious examples of which he has adduced from the works of Sir Theodore Mayerne; though, the author also remarks, superstitious practices ought not in every instance to be disregarded, since the rust of Telephus's spear, mentioned by Homer as a cure for the wound it had inflicted, was undoubtedly a useful remedy.

The ambiguity of language, is illustrated by the application of the terms *scurvy* and *yellow-fever;* and on the latter subject the author enters into an elaborate discussion, founded on observations deduced from very extensive researches; the chief object of which is to prove that what is properly yellow-fever, is a contagious disease. In force and precision, and methodic arrangement, his arguments cannot be excelled, and they must bring conviction to all who admit the data on which they are founded; but, we think, before this question can be decided in a manner that will be generally satisfactory, it must be first determined what is to be considered as yellow-fever; or else similar arguments to those used by Sir Gilbert Blane may be brought to show that that disease is not contagious. His data are derived from a division of the fevers of hot climates into three classes: the *endemic*, arising from marsh effluvia; the *pestilential* or *malignant* disease, or *typhus icterodes* of Sauvages and Cullen, from human effluvia; and the *sporadic.* The second, Sir Gilbert Blane states to be yellow-fever. Now, other physicians will assert, that it is the first which should be thus termed; and, for proof, refer to that which raged in the United States in 1794, 1797, and 1800, and especially at New-York in 1805, which was by general consent termed yellow-fever; and there can be but little or no doubt of its being caused by marsh effluvia. Rush at last abandoned the notion that it was propagated by contagion, although at first an advocate of that doctrine; and few men have been more cautious in judgment than him, and certainly not many better qualified to form a correct decision.

The observations and remarks on the fallacy of testimony, are too multifarious to be even alluded to in a concise manner: we can only say, that the reader will find amongst them many useful hints and judicious reflections, particularly valuable at a time when there exists a sort of rage for acquiring what are called new facts, instead of a disposition to meditate on those which are already known, and which have been established by the testimony of successive ages.

Before we part with the reader on this occasion, we have some apologies to make, and some remarks to adduce, that we particularly desire to impress on his mind. The first relate to the extent to which this article has been prolonged, chiefly by the minuteness with which we have examined some parts of the work to which it relates, and the passages introduced being somewhat adventitious to the subject under immediate consideration: we should add, too, any part of our examination which he may probably think bears a tone of undue severity. We request him to reflect on the interesting nature of the subject here discussed, and on the great importance it is to the welfare of our art, that no principles should be formally and systematically proposed as valid bases for our reasonings, that are not well defined in their character, and substantiated by indisputable arguments. For the rest, our desire to give a perspicuous view of the object of the work must serve as our excuse.

With respect to the question of the propriety of considering this work as a treatise on Medical Logick, even as far as relates to physiology, we have shown that we cannot form a determined judgment, generally, because we have not been able to comprehend the author's meaning on some occasions; though, on others, we have considered his opinions to be hypothetical, consequently, not proper for the principles of a system. It was from the difficulty just alluded to, that we were led to express so earnest a desire, on a former occasion, that the reader should peruse the work itself: otherwise, we fear, he may attribute that difficulty to dullness in us, rather than to real obscurity in the author. But we have other reasons for recommending this work to his attention. It has here been examined more expressly in its medical relations: we have, consequently, passed slightly over, or wholly neglected, what we believe to be the best and most useful parts of it,—the many good moral reflections it contains; we should add, too, many physiological observations, adduced as illustrations of given principles, which we could not apply in the manner the author has done, and therefore neglected to notice them. But it is the dispersed moral reflections that we have regarded with most pleasure: these form a series of traits of the medical character, calculated to exalt its dignity, in a scientific and moral point of view; and, when possessed, will render the physician really qualified for the practice of an art *Deorum immortalium inventioni consecrata.*

---

***An Enquiry, illustrating the Nature of Tuberculated Accretions of Serous Membranes, and the Origin of Tubercles and Tumors in different Textures of the Body.*** **By JOHN BARON, M.D. Physician to the General Infirmary at Gloucester. 8vo. pp. 307; with Engravings. London: Longman and Co. 1819.**

ON noticing this work on a former occasion, we stated it to be our intention to defer the particular examination of it, until we could comprise at one view the whole of the observations and opinions of the author on the different subjects to which it relates; and since he has not, in the present volume, adduced his general conclusions,—and, indeed, many of the facts on which the theory here advanced is founded,—such a measure would be prudent, both with respect to our ability to give an accurate exposition of his opinions in a concise form, and to the critical judgment we may be disposed to pass on them. But, the highly interesting nature of the facts here related, and the new views the author discloses of a series of objects previously but little attended to, or very inaccurately discerned, have appeared, on further reflection, to claim our immediate attention.

We shall endeavour to overcome the difficulties arising from the causes to which we have alluded, and give the reader a clear idea of the grand traits of this work, rather than a minute and regularly-progressive analysis of each particular part.

As the disease which seems to have led the author into the train of researches here disclosed, has not been much attended to by the generality of pathologists, we shall first adduce some observations and corollary remarks, that will serve, in some degree, to enlighten the way before us.

A tuberculated state of the serous membranes of the abdominal cavity, is an affection of such frequent occurrence, that it must have been witnessed by every pathological anatomist of at all extensive observation; but, from this disease not being accompanied by any strongly-marked symptoms in its first stage, it has not then engaged much of their attention; and, in its more advanced state, its origin has probably been too commonly confounded with its consequences. Many authors have given more or less comprehensive histories of it; but, to avoid a multitude of useless references, we shall confine our attention to the remarks of an accurate observer, of sound and cautious judgment; the luminary of modern physiology; and an excellent pathologist, of extensive observation, especially in diseases attended with organic lesion:—Dr. Pemberton, Bichat, and M. Broussais.

This malady is well described by Dr. Pemberton, in his treatise on Diseases of the Abdominal Viscera, under the head of Chronic Inflammation of the Peritoneum: for this he considered to be its origin. We may therefore save much time and space, by referring the reader to that classical medical work, for its history, symptoms, and the more general characters of the morbid appearances after death.

The observations of Bichât chiefly relate to its earlier stage: he first speaks of it as "chronic inflammation, with the production of a multitude of little whitish tubercles;" but, in this case, as in almost every other, Bichât advanced nothing positively of the truth of which he was not well assured, and which subsequent observation has not tended to confirm: he therefore, in the ensuing page, corrects the above opinion respecting its origin, by saying, on comparing the serous and cellular membranes, "there is a disease of the serous surfaces which we do not see in the cellular system: it is that slow inflammation of which I have just spoken,—a disease which we should rather arrange in some other class than that of the phlegmasiæ, and which is especially characterized by the production of the little tubercles which accompany it. Those authors who have not paid sufficient attention to this malady have denominated it chronic enteritis in the peritoneum, latent inflammation in the pleura, &c. It does not affect the subjacent organs except in the latter stages, when it is propagated by the medium of the cellular tissue; it has its seat exclusively in the serous membranes, and may be an affection peculiar to those parts, just as miliary eruptions are to the cutaneous, and aphthæ to the mucous, surfaces."*

* *Anatomie générale. Système Sereux.* Art. ii. § i.

M. Broussais has particularly described two cases of this disease in its last stage:* he notices it also, in a general manner,† in its primitive character. He considers it to depend on chronic inflammation affecting the *white capillary vessels*, and that this is not seated in the serous membranes themselves, but in the subjacent cellular membrane. Inflammation and thickening of the former texture, he considers to be an accident; and its elevation into tubercles of the form of hydatids, as a consequence of the effusion beneath them;‡ and the matter thus formed he has found to vary in consistence, from difference in the character of the diseased action, from a limpid fluid to a substance of cartilaginous hardness.

We marked out in a particular manner, the expression, *inflammation of the white capillary vessels*, in the above paragraph, because this relates to a very important part of the doctrine of M. Broussais. We cannot here enter into a detailed exposition of it; it must suffice for us to say, that, with Bichât, and many other eminent modern pathologists, he considers inflammation to vary from irritation only in degree, with regard to its nature; but that an important distinction respecting it is, whether it has its seat expressly in the sanguineous vessels, or in those which ordinarily, and even when inflamed, do not admit red blood except in a very partial manner; or whether it affects both those systems of vessels. It is inflammation of the white capillaries, or lymphatic vessels, that gives rise to tumors in general. This notion may appear merely hypothetical, when contemplated without any observations for its support; but it is rendered very plausible by the author's arguments.

The application of the above citations will be discerned as we proceed; and now we will commence our analysis of the work of Dr. Baron.

The author's history of the disease under immediate consideration may be passed over without inconvenience, by referring to the description given of it by Dr. Pemberton, excepting those observations which are peculiar to himself, or which bear a particular relation to his views respecting its nature and origin. The principal of those are, the distinctness of the tubercles from the organic lesions dependant on inflammation, which, as the author expressly says in one case, were "distinct in all their characters from the tuberculous disease, and manifestly had been formed at a much later period." Some of the tubercles he observed to be circular and pendulous, varying in

* *Histoire des Phlegmasies Chroniques*, tom. ii. Obs. 56 et 57.

† *Examen*, &c. p. 305.

‡ This notion, we may remark, accords with that adopted by Prof. MANGILI to explain the origin of the cysts, ordinarily termed hydatids, formed in the uterus. See the Sketch of the History of Medicine, in the forty-first volume of this Journal.

size from a pin's-head to that of a Spanish hazle-nut; some contained a soft, curdy, yellowish, matter; others were of a cartilaginous hardness and texture; and many were filled with a more or less limpid and colourless fluid; and some, by coalition, had formed tumors presenting great varieties of character. Similar appearances were frequently found in the peritoneum, lungs, pleura, and pericardium, of the same subject. Of the symptoms of the disease during life, we shall only notice a remarkable want of the nutrition of the body in every instance; the occurrence of a swelling of the lower extremities, often confined to one side, during the course of the disease, in several cases; extreme uneasiness in the stomach and bowels after taking food, or the ejectment of it by vomiting; and a sense of great weight in the intestines, which one patient compared to bullets appended to every convolution of them. Let us then, by way of summary, again observe, that the distinctness of the tubercles from the inflammatory affection; their hydatidical character; the signs of their progressive changes into tumors more or less dense, and even of a cartilaginous texture; and the existence of similar morbid appearances in parts of different species of structure; were the principal circumstances that led Dr. Baron to form his peculiar opinions respecting the nature of this particular affection; and subsequently, by the extensive and novel views his progressive researches continued to disclose, to a new theory of the origin of the greater part of the tumors witnessed in the human body. Let us now examine his particular opinions, and his arguments in favour of the theory to which we have alluded.

After some general objections to the too comprehensive manner in which inflammatory action has been brought forward in explanation of the origin of organic lesion, by the greater number of modern pathologists, Dr. Baron adduces Dr. Carmichael Smith's description of the symptoms characteristic of inflammation of diaphanous membranes, as "very accurate;" and he also remarks, that he was "induced to quote from the writer in question, because he was the first to introduce scientific and precise views into enquiries of this kind." On this point we must express an absolute dissent from the opinions of the author: we consider that description, as relates to inflammation of diaphanous membranes generally, to be vague in most respects, and erroneous in many important circumstances. Dr. Smith says, "we are certain that it (inflammation of diaphanous membranes) is attended with great pain, and with a high fever:" and the account he gives of its terminations, applies, as well as the above remark respecting its symptoms, solely to the more acute and intense forms of inflammation.

As Dr. Baron decidedly opposes the generally-received opinions respecting inflammation, or rather neglects even to notice that form of it commonly termed *chronic*, it is much to be regretted that he has not given, precisely, his own description of the symptoms that we may be allowed to consider designative of the presence of that species of morbid action. In identifying his opinions with those contained in the paragraph he has cited from Dr. Smith, he will, we believe, have to encounter the objections of by far the greater proportion of the best pathologists; for Dr. Parry's opinions respecting the forms of disease constituting the different varieties of inflammatory action, are now almost generally admitted. The author, on the contrary, assumes the proposition to which we have alluded, as a basis for his arguments against the opinions generally entertained respecting the inflammatory nature of this tuberculous disease of the serous membranes: "it exists (he says) without acute pain and high fever; and the appearances observable on dissection, do not accord with those which all the most *accurate* anatomists enumerate as consequences of inflammation."

We cannot designate the anatomists to whom the author alludes, because he has not given a reference to any of them, excepting Dr. Smith and Bichât, whose opinions on this subject we have already adduced. The most forcible objections Dr. Baron has produced against the inflammatory origin of the disease under consideration, are certainly his own observations made on dissection of the victims of that malady. It does from them appear, that the tuberculous affection did arise, in some cases, from a variety of morbid action differing from that of inflammation; unless we suppose inflammation had existed, and that all the other signs of it had disappeared before death. But here we advert to suppositions that would be unwarrantable, had not the most accurate and experienced observers described a similar disease, which they consider to have essentially originated in chronic inflammation; since it appeared to come on suddenly, from evident causes, in persons previously in good health, in whom no organic lesion could be suspected to exist, and it was attended from the onset with evident signs of inflammatory action. Dr. Baron, however, says, "Although pain, acute or otherwise, had been felt at some period of the disease by all the patients, it does not in any of them appear to have been such as results from any well-ascertained inflammatory affection; and we have just given proofs that the change of structure, both in the peritoneum and the mesentery, had proceeded to a great extent before inflammation came on. I think, too, we have the same thing demonstrated by the occurrence of effusion, in three of the cases, subsequent to the existence of all the early symptoms of the disease."

The objections that may be made to this statement by the partizans of the opposite doctrine, are, that the tubercles were the results of *chronic* inflammation, (the symptoms of which are often very obscure, and which is frequently not attended with much pain,) that may have persisted or subsided after these tubercles were produced; whilst the subsequent inflammation, to which Dr. Baron alludes, was an accident, similar to what occurs, sooner or later, in all cases of morbid change of structure of the soft parts. It is worthy of remark, too, in connexion with the opinions of M. Broussais, which we have already noticed, and with those of Haller respecting the origin of cystoid tumors in general, which we shall presently refer to, that inflammation of the cellular tissue is not attended with much pain, nor with constitutional disturbance, unless the inflammation be intense in degree, or seated in parts of a dense and unyielding structure,—as in those confined by a strong fascia, &c.: and thus we commonly find the pain and general disturbance of the system subside immediately after the division of the fascia of the hand and fingers, and of other parts, by the scalpel, in cases of inflammation affecting the cellular texture beneath those membranes.

We shall transcribe one of the cases which Dr. Baron states to be particularly adapted to illustrate his opinions of the origin and nature of the disease under consideration. This assertion, and its conciseness, have led us to select it from the many related in this work.

"Priscilla Bullock, æt. 17, Feb. 5, 1818. Complains of pain and tenderness of the belly, with frequent cough, and quick and laborious respiration. The appetite is very bad, and what food is taken is speedily rejected, after violent retching. The bowels are loose, and she has a general sense of uneasiness about them. Pulse 130. The skin is hot, and for the most part parched; except at night, when she has cold sweats. The tongue has a bright red colour. The lips are dry, and the cuticle is frequently peeling off. She complains also of pain about the shoulders and chest.

"This girl's symptoms are reported to have come on about ten weeks ago, after being exposed to cold and rain. The looseness began at that time, and it has continued ever since. About a fortnight ago she was attacked with measles. To that attack she ascribes her cough and the uneasiness about the chest.

"I pronounced this to be an affection of the peritonæum, and probably also of the lungs and pleura, like those of Tandy and Aldridge. A complete removal of some of the most distressing and characteristic symptoms, led me at one time to doubt the accuracy of my diagnosis. She was repeatedly blistered; and the irritable state of the stomach and bowels was so effectually obviated by chalk, and catechu and opium, that both the vomiting and looseness left her about a fortnight after she came to the Infirmary. The velocity of the pulse, too, was very much diminished; and so were the cough and difficulty of breath, ing. The appetite improved, and she certainly gained flesh. This circumstance convinced me, that, though the peritonæum might be affected, the mesentery could not have suffered so much as in the other cases.

"She continued improving till the 1st of April. Then came on more cough, with heat of skin, and a quick pulse and pain about the chest and belly; but it was especially severe in the temples and the forehead. She was bled by leeches on the head, and purged. She nevertheless fell into a state of stupor; the pupils of the eyes became dilated, and a rigid and spasmodic action of many of the muscles took place. During this state, the disorder, both of the thoracic and abdominal viscera, seemed to give her no uneasiness. She was bled at the arm; the head was shaved, and both temporal arteries were opened; and purgatives and blisters were used: but all did no good. She died on the 7th.

"I next day examined the body. The abdomen felt hard and tense, but it was not protuberant. Its peritonæal lining was universally thickened and tuberculated. The tubercles were very numerous, many of them being circular and pendulous. They varied in size, from that of a pin's head to that of a Spanish hazel-nut. The latter contained a soft, curdy, yellowish, matter. Others were of a cartilaginous hardness and texture. On the surface of the intestines they were numerous, and small, and distinct."

In his reflections on this case, Dr. Baron observes,

"The case of Bullock affords the only example I have ever seen, of a complete interruption to the progress of the disease, after its peculiar characters were apparent. The account of the dissection, in some measure, explains this circumstance. The mesentery was not completely involved in the disease; and the adhesive inflammation seems to have come on subsequent to the existence of the tuberculous disease, and to have destroyed her before the other affection had run its usual course."

After a further development of the arguments we have already noticed, against the doctrine of the inflammatory origin of this disease, the author advances a proposition, respecting which the reader must suspend his judgment until he has become acquainted with the facts on which it is founded. It is by them rendered, at least, plausible; and the author states, that he has yet many others to produce in its favour. These we wait for with much anxiety; since there are some points in his theory that still require the support of facts to make it satisfactory. After noticing the opinions of Bichât on the tuberculous affection above described, he says,

"There is reason to believe that this writer has fallen likewise into error, in describing this disease as peculiar to serous membranes, Tubercles exist in almost every texture, and their origin and essential character will probably be found to be the same, wherever they are discovered."

We shall proceed to the author's application of this proposition, after we have adduced his opinions respecting the origin of the disease which has already engaged our attention. He says,

"We have not been able to show where this disease really originates, though it is perhaps now apparent where it does not. Nothing that is known, either of the physiology or pathology of the sanguiferous system, seems capable of explaining what we are now in quest of. We must therefore direct our attention to another part of our structure."

It is to the absorbent system to which the author adverts; though he has not in the present treatise advanced any thing positive, in the way of direct argument, on this subject. We shall, however, adduce what he has stated.

"This enquiry has hitherto been confined to the symptoms and consequences of tubercles in the peritonæum; but the dissections have proved the existence of the disease, not only in other membranes of the same class, but in other textures. They have been found in the lungs and in the pleura; and it will afterwards appear, that they may exist in almost every texture of the body. Their origin must therefore be connected with some one of those elementary parts of our frame, which are diffused through the body, and enter into the composition of every organ. This is the case with the sanguiferous, and so it is with the nervous and absorbent systems. Disorders of the latter are supposed to occasion many of the symptoms of a numerous and most unmanageable class of diseases. To this class may perhaps be referred the complaint under consideration; and we are now to see whether, by fact or analogy, we can trace the morbid appearances so far as to enable us to give a satisfactory account of their origin."

Passing over the question of the propriety of thus *searching* for principles to serve for the overthrow of an existing theory, and the establishment of a new one, we stop to point out an omission in the above enumeration of the different structures of the body, universally distributed, that a little surprises us,—we mean the *cellular tissue;* especially, too, when it was the opinion of HALLER, that cystoid tumors originate solely in this tissue: and those pathologists who agree with him, will not fail to bring forward in their support the very forcible arguments in favour of their opinion furnished by Bichât's treatise on the texture we have mentioned. It was this consideration which led us to refer to the observations of M. Broussais, at the commencement of this article, as illustrations of the opinions to which we have alluded.

Dr. Baron refers to the history of a case related by Sir EVERARD HOME,* which, he considers, supports his opinion. He,

* See page 28 of his treatise on Cancer.

indeed, says, " As far as one case goes, the evidence of what has been quoted is very conclusive; and, when it is examined in conjunction with the facts in another part of this enquiry, it affords, at least, fair reason for believing, that the opinion to which I incline is not destitute of foundation."

We do not regard this in so convincing a manner; but, as the settling of this point is not of essential importance in the author's theory of the origin of tumors in general, which is the most interesting part of this work, we shall pass over it, to the curious facts on which that theory is founded, and respecting which we have already bespoken the reader's attention. These are the various transformations of *hydatids*, using the author's appellation, from mere cysts containing a more or less limpid and colourless fluid, into tumors possessing different characters, especially as relates to their degrees of density, from a mellicerous consistence to cartilaginous hardness and texture. This, it should be remarked, had been previously stated, in a partial manner, as we shall hereafter more particularly show, as a matter of conjecture, rather than of precise observation. It was Dr. Jenner, the author says, who first pointed out this occurrence to him, and who has demonstrated the fact by experiments on rabbits and some other young animals.

" He found that, by feeding them with some kinds of food, the liver soon became studded with hydatids; and, by examinations at different times, he was able to trace the gradations already mentioned, from the first inspissation of their contents and thickening of their coats, to their final conversion into tubercles of varying size and hardness. In the same liver he has also repeatedly found every gradation, from compact caseous-looking matter, to the limpid contents in their first stage."

Some of the facts above alluded to were communicated to Dr. Beddoes, and were published by him in his work on Factitious Airs; but they have failed to engage the attention of pathologists in general.

Dr. Baron has witnessed similar instances of morbid transition in the human body; and, what is worthy of particular remark, cysts containing matter having diversities of character, were found to spring from one small root or pedicle hanging into the abdominal cavity like a bunch of grapes. Others exhibited a diversified series of concentric laminæ, like many urinary calculi.

The author also adduces some cases from De Haen* and Boerhaave,† and other authors, illustrating also this species

* *Ratio Medendi*, t. i. c. xx. part vii. c. xvi.; et *De Hydrophe Cystico et Hydatidibus.*

† *Epist. Anat. ad Fred. Ruysh*, p. 73 and 82; and his history *Atrocis Rarissimique Morbi.*

of transformation. He then recurs to arguments against the doctrine of the inflammatory origin of tubercles; and again advances, in general terms, his opinion that their formation may be more satisfactorily ascribed to the absorbent than to the sanguiferous system; and that *their essential character is probably the same, in whatever texture they are seated.*

We cannot ourselves form any plausible conception how a cluster of tubercles, hanging from a pedicle like a bunch of grapes, can originate from disease of the absorbent vessels. We will leave this subject to the reflection of our readers, and proceed to the author's application of the proposition last advanced, to the explanation of the origin of cancer, fungus hæmatodes, tuberculated sarcoma, and fatty tumors. He first examines, in a particular manner, the cases related by Sir Everard Home* of the existence of hydatids in the breast with cancer; endeavouring, of course, to show, that the existence of hydatids should be considered as the origin of the disease, not as an accidental occurrence. This notion, it will be recollected, was some years since advanced by Dr. Adams, who, it appears, derived the ideas that led to it from the paper of Dr. John Hunter on Hydatids, as Dr. Baron points out. It should be remarked, that Dr. Baron did not become acquainted with this coincidence between his opinions and those of Dr. Adams on this subject, until the present volume had nearly passed the press; but he stops to adduce some remarks on the particular development of this doctrine, given by the last named physician. We are not disposed to advocate the notions of our late friend on this subject; but the regular examination of Dr. Baron's theory leads us to notice one of his objections to them, on which he seems to lay particular stress: this applies to the opinion attributing cancer to the *living state* of the hydatid, admitting that hydatids are animalcules: he has shown, he says, "that it is to the loss of the hydatidical character altogether, and the transformations of these bodies, that the morbid appearances in this, and in many other diseases, are to be referred." This distinction is not perfectly intelligible to us; and we wish the author had entered into an explanation of it, because it involves a point of much interest in his theory.

If hydatids are animalcules, and Dr. Baron is inclined to believe them such, we cannot suppose the changes described by him can take place in them after their death. How can a limpid fluid in a dead animal become changed into a substance of cartilaginous texture? If they are only a morbid formation of, or from, the part in which they are seated, what is the precise state, in their progress from a cyst containing a limpid fluid to a

* See page 108 of his Treatise on Cancer.

pultaceous or cartilaginous mass, at which they may be considered to constitute cancer? This the author has not explained.

The author's arguments in favour of his doctrine of the hydatidical origin of the various species of tumors we have enumerated, will not, of course, admit of being at all represented in so concise an abstract as our limits confine us to: we can only say of them, that they are highly ingenious, and very plausible as relates to many points; but we are not at all inclined to give our assent to his theory generally. Yet it has hardly the air of due candour and liberality, to make such a remark without having given an exposition of the author's particular arguments, or of the reasons for our dissent. The latter would extend too far this article, for we have yet much to adduce by way of analysis of the work: we shall, then, choose a subject, on which we will give the whole of Dr. Baron's discussion on one case; and, with respect to the manner in which it favours his doctrine, as well as its adaptation to our limits by its conciseness, we perceive none of them more appropriate for transcription than that on a case of fungus hæmatodes, related by Mr. HEY.

"Among these diseases I have mentioned the fungus hæmatodes. This name, it has been justly remarked, is calculated to mislead. Mr. Hey, when he applied it to the formidable case of that description which came first under his observation, was guided by an appearance, which, perhaps, ought to be considered rather as an occasional than a constant symptom; a symptom which is only seen in parts previously diseased, whose morbid texture and condition have been altered by external violence or other causes.

"These things must be kept in remembrance, otherwise it may be supposed that I have been pursuing unfounded analogies, and incautiously drawing inferences from diseases which are at variance with the doctrines advanced. It is, comparatively speaking, of little consequence what name we assign to disorders, provided that name be well defined; and the reader will perceive that I have been much less anxious to establish uniformity on that point, than to gain a true knowledge of morbid appearances, without which all attempts at amending our nosology must be useless, if not pernicious.

"When, therefore, the disease in question gets into that state which accords with and justifies its name, the fungus is to be looked upon, not as a specific and essential symptom, but an accidental one, determinable altogether by something peculiar in the course of individual cases. Thus, if we trace their progress, we in general find tubercles or tumors, at first small and distinct, advancing with different degrees of acceleration, and communicating to the touch different sensations, according to the predominant character of their contents. These, and all other tumors, during their growth, must have a great effect upon the surrounding parts; and, while they are in a diseased state, should the skin give way, in consequence of external violence or any other cause, we sometimes see those frightful, bloody, fungated sores, which first suggested the name of the disease.

"This progress will be best illustrated by attending to the course of the first case described by Mr. Hey. The disease commenced in the form of a small movable swelling on the inside of the right knee, not far from the patella. It does not appear to have been occasioned by external injury, but external injury much modified its subsequent nature. The man, half a year after its appearance, fell against a stone, and hurt his knee; then the tumor increased till it became the size of an egg. It remained in that state till two months before he was admitted into the Leeds Infirmary. He then fell from a piece of wood, and violently bent the diseased knee, but did not strike it against any thing. 'The tumor began immediately to enlarge; and, within a few hours, extended half way up his thigh, on the inner side of the limb. About a fortnight after this last accident, the skin burst at the lowest part of the tumor, and discharged some blood. A dark-coloured fungus, about the size of a pigeon's egg, appeared, and remained at this part. A few weeks after the appearance of this fungus, the skin burst in another part of the large tumor, and discharged some blood. From the fissure arose another fungus, which had increased, in the course of the last week, to the size of a small melon, and now measured eight inches over, between the opposite parts of its base. Blood frequently issued from the base of this fungus, chiefly when the man hung down his leg.'

"Now let us attend to the morbid appearances of this disease, before it has proceeded so far as in this case, or its nature been changed by violence or other means; and I think it will incontestibly appear, that the remains of the hydatidical character may be more distinctly traced in it than in almost any other affection.

"Mr. Hey's third case was a tumor in the mamma. 'It was perfectly distinct from the surrounding adipose membrane; having no other connexion with it than by that cellular substance which universally connects the contiguous parts of the body. When divided by the knife, it had the appearance of a diseased glandular substance, intermixed with small cavities containing a viscid or gelatinous serous fluid.'*

"The fourth case was of the same description. 'The tumor adhered in part to the mamma, and had the appearance, when divided, of a diseased glandular substance, interspersed with three or four cysts, containing a viscid serous fluid.'"†

The author has selected from the most esteemed writers, several histories of the different species or varieties of disease to which we have already alluded, and examined them, generally, in a more detailed and more minute manner. With respect, therefore, to his mode of pursuing this enquiry, there can be but one opinion, whatever degree of assent may be given to his theory.

---

* Vide *Hey's Surgery*, p. 267. † *Ibid.* p. 271.

The questions respecting the origin of hydatids; whether or not all the cystoid tubercles referred to in this enquiry have the same origin; and what is the nature and seat of the morbid action from which they originate; do not, it must be evident, affect that part of the author's theory under immediate consideration.

We have now given a view of the principal traits of this work, and it is such as will, we believe, convey to the reader of this abstract accurate and tolerably comprehensive ideas of them; that is, as far as relates to observations and theoretical conclusions: the particular reasonings of the author must, of course, be sought for in the original, as they cannot be comprised in the limits to which we are here confined.

On taking a revisal of our analysis, we have, however, discerned that one point, a glimpse of which was given on a former occasion, (page 407,) yet remains to be rendered more clear,—which is, the author's observations respecting the formation of tumors from tubercles.

By *tubercle,* Dr. Baron would signify "those disorganizations that are composed of one cyst, whatever may be its magnitude, or the nature of its contents;" and by *tumor*, "those morbid structures that appear to be composed of more than one tubercle." Their particular and general formation is then thus explained: "It is probable that all tubercles, wherever situated, and of whatever substance composed, were, at their commencement small vesicular bodies with fluid contents."

A single hydatid, then, when transformed, will give rise to one tubercle, which may be either pendulous from, or embedded in, a part; and several may be more or less closely dispersed on the texture from which they arise. Hydatids also sometimes grow in clusters, somewhat like bunches of grapes, and thus hang into the different cavities of the body. Sometimes small hydatids grow from the outer or inner surface of larger ones, or float within them. Hydatids thus pendulous from, or dispersed in, a part, will become tubercles, and increase in size or in number until they encounter each other; they will then coalesce, and thus form a tumor. As any particular group of hydatids may arise from parts of diversity of texture, as well as from diversity of morbid action, this group will present tubercles of various characters, some containing fluid, others solid matter, &c.; and this variety will be imparted to the tumor thence formed. Lastly, those tubercles which are constituted by hydatids arising from the internal or external surfaces of others, will present an appearance of concentric layers, similar to what is seen in many urinary calculi. Instances of all these varieties are given by Dr. Baron.

We should notice in a particular manner the author's application of his observations to vomica of the lungs. He considers that the cases of this affection placed by SAUVAGES in his class *anhelationes*, but which CULLEN considered solely as the consequence of inflammation, arise from a cyst being formed in the organ, similar in nature to those above alluded to; and he adduces some cases in support of his opinions, where the patients certainly did not present well-marked symptoms of inflammation of the lungs until after the bursting of the vomica, or at least until a short time before this took place. Inflammation, Dr. Baron remarks, generally comes on after the discharge of the contents of the cyst, probably from the admission of air to its cavity, and then *real pus* will be expectorated, and hectic fever ensue.

We, on a former occasion, gave our reasons for passing over unnoticed the author's history, &c. of the tuberculous affection of the peritoneum: we should also apologize for not considering, in a particular manner, his observations on its treatment. The principal reason is, that we thought it best to devote the space we could assign to our review of this work, to the most interesting part of it; another is, that medical aid has not hitherto been of much evident utility. We may, therefore, confine ourselves on this subject to the following extract, and some detached observations.

"By keeping the alimentary canal in a healthy condition; by freeing the mouths of the lacteals from obstructions; by apportioning the diet, both in quantity and quality, to the wants and powers of the system; by avoiding every thing whereby these powers may be either too much excited or exhausted; by applying suitable means to subdue such local affections as may either have existed, or may arise, in the course of the complaint; by attending closely to the state of the skin; by promoting the healthy action of the extreme vessels; by bathing of various kinds, by frictions, and well-regulated and well-timed exercise; we may often witness the most gratifying results, in such persons as I have alluded to, (those in whom the functions of the digestive and nutritive systems are falling into disorder, which the author seems to consider as the general origin of the disease,) instead of being compelled to watch the progress of fatal disorganization, which probably would have arisen, had less rational and comprehensive views directed our practice."

Bleeding was occasionally useful, to relieve inflammation as it arose, or as it became more intense. Mercury was found to be hurtful rather than beneficial. Heat at the stomach was sometimes alleviated by the mineral acids, and gastrodynia by the oxide of bismuth. But the most interesting remarks on this subject, are those relating to the disappearance of morbid growths under the influence of nausea, caused by medicine, as emetic tartar, squills, digitalis, &c.: and, by a method proposed by Dr. Jenner, the notion of which he derived from some observations on brute animals,—of using a highly nutritious diet, after having maintained sickness by medicines for a considerable period. Dr. Jenner says, " Rabbits taken in a state of health, and put upon innutritious diet, soon began to be diseased, and hydatids sprung up upon the surface of the liver. These were thrown off, even when advanced to the incipient stages of tubercles, by restoring the animals to health by means of plenty of the most invigorating food."

Since we have, as we proceeded, in the foregoing analysis, expressed our dissent from all those propositions advanced by the author that we think to be disputable; and, on the contrary, stated positively those which appear to be well determined; it is not necessary for us to give a summary of our opinions respecting the points that seem to be established in this work: only this should be borne in mind by the reader of this abstract, that, much that here appears objectionable, may be so only from his not being acquainted with the whole of the facts on which the author's theory has been founded, and from the want of the greater part of his arguments in its support. On the other hand, a single perusal of the work itself, will, perhaps, lead to opposite sentiments: such, at least, was the case with us. The novelty and interest of the facts, the methodic manner in which they are arranged, and the boldness and rapidity of the reasoning on them, more than once induced us to join the author in positions which we have since been led to desert,—whether by a want of proper firmness of mind, or a severe mode of reasoning, is not for us to determine. These remarks will, however, serve as an apology for any unusual caution that may appear in this examination. An excellent critic, in his *beau ideal* of a Journalist, said, " *plus la matière sera importante, plus il se montrera difficile;*" and it was precisely this sentiment of DIDEROT that here influenced our conduct. We, then, conclude with earnestly recommending this addition to the science of pathology to the assiduous attention of our readers; and with stating that, when we are in possession of the whole of the author's observations and reflections, we shall assume either a more determined tone of assent to, or of dissent from, the new theory he has here advanced.

